# Health Policy Analysis Requires Attending to Institutions: Comment on "Modelling the Health Policy Process: One Size Fits All or Horses for Courses?"

**DOI:** 10.34172/ijhpm.2023.8085

**Published:** 2023-08-07

**Authors:** Patrick Harris

**Affiliations:** Centre for Health Equity, Training, Research & Evaluation (CHETRE), Part of the UNSW Sydney Research Centre for Primary Health Care & Equity, A Unit of Population Health, South Western Sydney Local Health District, NSW Health, Ingham Institute, Liverpool, NSW, Australia.

**Keywords:** Health Policy, Policy Theories, Institutions, Power

## Abstract

Powell and Manion present an important review of reviews about health and policy research. They zero in on theories of the policy process as the most likely to unearth what is really going on in health policy. Here I contend that their analysis insufficiently attends to institutions. Powell and Manion situate ‘institutions’ (with ‘Ideas’ and ‘Interests’) as a ‘health policy process models’ that ‘tend to’ list factors rather than connect them. Rather, I show how there is a rich history of considering institutions in the political science literature that is under considered by Powell and Manion. By necessity for a Public Health audience I quickly pull back the covers on ‘rigour’ and ‘causation’ to demonstrate what is ‘fit for purpose’ in rigorous institution focussed policy analysis. I conclude by arguing how institutionally focussed public health policy analysis is vital for understanding and addressing heath inequities. That focus necessitates research that provides better, explicit, conceptualisations of power in health policy: especially drawing out the roles played by structure and agency. I offer some recent examples.

## Introduction

 I thoroughly enjoyed Powell and Manion’s^1^ detailed consideration of health policy analysis. Their editorial adds to the growing literature that demonstrates policy analysis as legitimate, and crucial, for health research. However, their analysis underemphasises institutions. In particular, the dynamic role of structure and agency is under considered.

 My response here is not to criticise, but to add to, their insightful analysis theories of the policy process. The main flaw in Powell and Manion’s analysis and argument is limited engagement with institutions. Their focus is theories of the policy process, using Weible and Sabatier’s^2^ introductory text. Once these theories are introduced, they then review the ‘health policy process’ literature for consideration of these theories, finding that few of these theories are used in that literature. This line of analysis is all well and good, and usefully demonstrates how a deeper engagement with cross-disciplinary scholarship is needed to add depth to health policy analysis.

 However, the argument falters in their conclusion about institutional factors tending to be static in health policy analysis, relative to theories of the policy process. I recognise that they take aim at the use of these factors in health policy process models as ‘lists’ rather than full explanations. But their under consideration of the dynamics of institutions means their analysis comes up short.

## Why Institutions Matter: Structure *and* Agency

 There is another foundational text about policy that emphasises institutions as the basis for understanding the essentials of policy. I urge readers to start with, or go back to, Howlett and colleagues’^3^ (updated in 2022 with a new title but same institutional emphasis) ‘Policy cycles and subsystems’ *before* reading Weible and Sabatier. Why so? Institutional analysis, looks, Howlett et al explain, to the structure of political and economic arrangements. These structures are famously described ‘rules of the [policy] game.’^4^ ‘Institutions,’ political scientists therefore claim, ‘matter.’^5,6^

 Howlett et al importantly explain that their disciplinary orientation, is Statist. That is, their core concern is with the State — formed around but going beyond government — as the main arbiter of policy. The State is an institution that is made up of actors (the people and organisations involved), ideas (the content) and structures (the rules and mandates that condition institutions). Statism is deeply connected to a whole body of political theory, institutionalism,^7^ which is based on shifting attention to the role of structure and agency in policy. Structures, in contrast to agency, focus on the conditions that ‘structure’ society by creating the rules and mandates that institutions tend to maintain. Change, if and when that comes about, occurs through the power of agency, for instance the role of individuals or groups in disrupting the status quo to create new rules and mandates. In this way, institutions embody both structure and agency, and a full and deep policy analysis recognises that distinction.^8^

 Not everyone emphasises this structure and agency differentiation. Peters,^9^ for instance navigates the history of institutional analysis to land on the same three ‘I’s’ (institutions, ideas, and interests) that Powell and Manion claim as static factors in health policy analysis. Similarly, Carol Weiss in her seminal work on evaluation evidence in policy, falls on the three I’s.^10^ As long as the dynamics created both by structures and agency are kept in full focus, then institutions clearly do matter for policy analysis.

 A quick text search for the term ‘structure’ in Powell and Manion’s text shows the word is absent. That absence has similarly been picked up in theories of the policy process.

 For example, in the political science literature scholars have taken theories of the policy process to task for limited unpacking of institutions.^11,12^ The ‘Big Three’^13^ — Multiple Streams Analysis (MSA), Advocacy Coalition Framework (ACF), and Punctuated Equilibrium Theory (PET) — have been especially accused of insufficient explicit attention on the structuring dynamics of institutions on policy as the ‘[formal and informal] rules…and social norms’ that constrain and shape the behaviour of agents and networks in the policy process (p. 120).^11^ That omission — aside from Ostrom’s Institutional Analysis and Development theory — leads to questions over whether theories of the policy process provide sufficient causal depth of synthesis and understanding about the structural rules and policy-making.^11,12^

 A quick diversion into what is meant by causation is necessary here. Powell and Manion dance around causation, using ‘rigour’ rather than the term ‘causal.’ That omission opens up a world of confusion for disciplines like Public Health that have a tradition of focussing in on a particular type of causal inference. Policy analysis belongs to the social sciences. The emphasis is complexity and conditions. Public health research has tended to take on a reductionist approach to causality that factors out complexity and conditions in the search for certainty. Neither is wrong, but both are different. Public health intervention focussed research falls into what Howlett et al term ‘research *for* policy.’ Policy analysis falls under what Howlett et al^3^ term ‘research *of* policy.’ By opening up the can of worms of ‘rigour,’ Powell and Manion risk conflating two very different, albeit complementary, analytic enterprises. Indeed, ‘rigour’ in most if not all of the studies reviewed by Powell and Manion concerns opaque or omitted reporting of data collection, analysis and use of theory. None of those papers uses terms like ‘causal certainty.’ Most support the widespread support in political science for using more than one theory of the policy process given the complexity of the object that is policy.^14^ Deductive theory testing to explain the reality of policy is, by and large, insufficient.^8^

 Thus far I have introduced the depth provided by institutional theory as a blind spot in Powell and Minion’s otherwise excellent editorial. Here I want to drill down into why, with a health lens, that omission matters. There is a raft of evidence that the health inequity is persistent and worsening. What is missing from the above analysis is a focus on the essential mechanism in policy systems that maintains those health inequities: power. Power and a structural focussed institutional analysis of policy go hand in hand, as I demonstrate shortly. Powell and Manion, however, only refer to power in their description of two health policy analysis sources.

 Powell and Manion suggest that a full policy analysis requires additional literature beyond theories of the policy process. My and my colleagues’ work — in the ‘determinants of health’ body of health policy identified by Powell and Manion — supports that position. Indeed, I have recently identified the point in my program where theories of policy process proved unable to capture the nuances of the policy system I was investigating.^8^ Ultimately, however, we have found that while additional literature helps explain the nuances of a particular policy being investigated or analysed, an explicit focus on power is necessary to draw out those nuances. Turning full circle to my points above, we have demonstrated how power in policy is agentic, ideational, and structural.^15^ Our body of work has provided increasingly sophisticated frameworks to health focussed policy analysis and evaluation.^16,17^ We have, for instance, shown how policy systems are set up and maintained (termed Path Dependencies) to create the conditions for increasing health inequities. Specifically, for instance- using a mix of theories to explain data — explained how power and governance are mutually reinforcing.^8^ That intertwining actively omits health equity from policy goals like ‘economic growth’ when equity is perceived to challenge the values underpinning those goals, such that pro-equity voices are not allowed a seat, voice or influence. Nevertheless we have also shown how the power of ideas, driven by actors to disrupt the status quo, can disrupt those entrenched power dynamics.^16^ Part of the challenge of this type of analysis is that change is often slow, incremental, multi-faceted and long term. Research funding, given its tight timeframes and particular interest in tight causal analysis of ‘what works,’ often structures out the research Powell and Minion’s editorial advocates. Institutionally centred analyses, however, provide for deeply nuanced explanations of policy that directly challenge Powell and Manion’s conclusion that the health policy analysis literature is ‘conceptually weaker’ than theories of the policy process.

 In conclusion, Powell and Manion provide an excellent introduction to the challenges of health policy analysis that is disconnected from the political science literature. However, they seem to be treading a fine line with their coverage of the theories of the policy process which risk under-playing the structural and agentic power of institutions.

## Ethical issues

 Not applicable.

## Competing interests

 Author declares that he has no competing interests.

## References

[1] Powell M, Mannion R (2023). Modelling the health policy process: one size fits all or horses for courses?. Int J Health Policy Manag.

[2] Weible CM, Sabatier P. Theories of the Policy Process. Routledge; 2018.

[3] Howlett M, Ramesh M, Perl A. Studying Public Policy: Policy Cycles and Policy Subsystems. 3rd ed. Canada: Oxford University Press; 2009.

[4] North DC. Five propositions about institutional change. In: Knight J, Sened I, eds. Explaining Social Institutions. The University of Michigan Press. 1995:116-131.

[5] March JG, Olsen JP (1983). The new institutionalism: organizational factors in political life. Am Polit Sci Rev.

[6] Hall PA, Taylor RC (1996). Political science and the three new institutionalisms. Polit Stud.

[7] Hall PA. Governing the Economy: The Politics of State Intervention in Britain and France. New York: Oxford University Press; 1986.

[8] Harris P. Illuminating Policy for Health: insights from a decade of researching urban and regional planning. Palgrave McMillan; 2022. 10.1007/978-3-031-13199-8.

[9] Peters BG. Institutional Theory in Political Science: The New Institutionalism. 4th ed. Edward Elgar Publishing; 2019.

[10] Weiss CH (1999). The interface between evaluation and public policy. Evaluation.

[11] Real-Dato J (2009). Mechanisms of policy change: a proposal for a synthetic explanatory framework. J Comp Policy Anal Res Pract.

[12] van der Heijden J, Kuhlmann J, Lindquist E, Wellstead A (2021). Have policy process scholars embraced causal mechanisms? A review of five popular frameworks. Public Policy Adm.

[13] John P (2003). Is there life after policy streams, advocacy coalitions, and punctuations: using evolutionary theory to explain policy change?. Policy Stud J.

[14] Cairney P (2013). Standing on the shoulders of giants: how do we combine the insights of multiple theories in public policy studies?. Policy Stud J.

[15] Harris P, Baum F, Friel S, Mackean T, Schram A, Townsend B (2020). A glossary of theories for understanding power and policy for health equity. J Epidemiol Community Health.

[16] Friel S, Townsend B, Fisher M, Harris P, Freeman T, Baum F (2021). Power and the people’s health. Soc Sci Med.

[17] Schram A, Townsend B, Mackean T (2022). Promoting action on structural drivers of health inequity: principles for policy evaluation. Evid Policy.

